# Bleeding Diathesis or Fabrication: Munchausen Syndrome

**DOI:** 10.7759/cureus.1339

**Published:** 2017-06-12

**Authors:** Syeda Naqvi, Raad Asadullah Khan, Chintan Rupareliya, Rida Hanif, Zeeshan Ali, Faiza Farooq

**Affiliations:** 1 Jinnah Postgraduate Medical Centre, Jinnah Sindh Medical University (SMC); 2 Department of Neurology, University of Missouri, Columbia, Missouri; 3 Medicine, Jinnah Sindh Medical University (SMC); 4 Psychiatry, Kings County Hospital Center

**Keywords:** munchausen syndrome, platelet, blood, fabrication, bron von munchaausen, cognitive behavioral therapies, functional disorders, bleeding diathesis, psychiatric disorders, psychiatry

## Abstract

A case history of an 18-year-old female with a diagnosis of Munchausen syndrome is presented with a literature review of this rare syndrome. We present this case because of the young age and the patient's overwhelming response to cognitive behavioral therapy. We recommend collateral history taking, exclusion of all possible etiologies and detailed briefing of family members as it plays a vital role to reduce the mental and financial suffering of the patient.

## Introduction

In 1951, Munchausen syndrome was first named by Asher after a German military man, Baron von Munchausen. He traveled from place to place telling fantastic tales about his imaginary exploits. Munchausen syndrome is unique among other factitious disorders as it includes extreme chronicity of sick role and seeking treatment and desired attention [1-2]. It is characterized by consciously feigning and fabricating symptoms to adopt a sick role.

## Case presentation

An 18-year-old unmarried young Asian female came through the emergency department (ER) with a history of hematemesis for last twenty-four hours. The patient reported that this was the third episode of hematemesis in one day. She was managed in ER for non-variceal upper gastrointestinal bleeding (UGIB) with fluid support and urgent transamine injections. Her blood pressure was 100/60mm Hg and pulse were 100 per minute. After stabilization, she was transferred to the inpatient ward for further investigation and management.

On examination, the patient was in acute distress due to her recurrent bleeding episode. She had elicited tenderness on palpation in right upper quadrant (RUQ) and left lower quadrant (LLQ) of her abdomen. Rest of the physical examination is unremarkable. Her weight was 45kg and height was 162.5cm. She had never been to school and her education took place at home by her family.

Previously, she had severe headaches with 8/10 in intensity for three months. She had a computerized tomography (CT) scan done two months ago for her headache in a different hospital where she was admitted due to her complaints of a headache and blurry vision. She further claimed that CT scan showed the blood clot in her head and she was taking a blood thinner (unknown anti-platelet agent) for it. The patient was unable to present the reports when a physician asked for it. She took a blood thinner which was available over the counter (OTC).

According to her, she had first episode of bleeding from the ear when she was two years old. She never had any bleeding episode afterward. She had menarche at an age of thirteen years and she had regular menstruation and reported the use of one pad per day for four days every month. For last three months, she had menometrorrhagia and she also had watery discharge along with it. Complete work up for bleeding disorders (bleeding time, prothrombin time, quantiative analysis of platelets) and serology (autoimmune disorders) ordered came back normal.

Later, the patient also mentioned about seizure episodes associated with her menses. She told that she had three to four episodes of generalized tonic-clonic (GTC) seizures, each lasting for 10-15 minutes. Her last event of seizures was in the emergency department. The event was not witnessed by anyone (physicians, nursing staff or family member). She experienced a severe postictal headache in the frontal region.

She got symptomatic relief in a headache with acetaminophen. Upon admission, baseline labs were ordered including complete blood count (CBC), basic metabolic panel (BMP), liver function test (LFT), complete bleeding profile, antinuclear antibody (ANA), electroencephalogram (EEG), electrocardiogram (ECG), chest X-ray (CXR), stool detailed report and urine detailed report was ordered. All her tests came back normal.

She again had an episode of upper gastrointestinal bleeding on the third day of admission. This time patient had fresh blood on her shirt. This event was concerning for the possibility of a qualitative platelet defect or a rare collagen disorder. She underwent qualitative analysis which did not show abnormalities on Adenosine 5’- diphosphate (ADP) and collagen test. The patient was evaluated by a gastroenterologist and had an esophagogastroduodenoscopy (EGD) which showed mild gastritis. In addition, a viral panel (Hepatitis B and Hepatitis C) and a CT scan of the abdomen and the chest were non-suggestive of any pathology.

The patient was given omeprazole oral tablets twice a day and was managed symptomatically. Next day she had blood in tears as shown in figure #1 which was an alarming symptom and raised the concern of haemolacria secondary to ophthalmological disorder. Ophthalmology consult was made and after ruling out every serious possibility like trauma, tumors, etc., she was put under close surveillance for haemolacria episode. On the same night, she bled again from eyes with each layer of bloody tears coming from eyes. She also mentioned blood oozing from nipples and vagina. Repeat physical examination was done and a bottle was found beneath her bed which she refused to give initially and kept insisting that the bottle has medications. Upon opening the bottle, a nurse found blood in it which she said she collected from her nipples. There was no mark of any fresh or dried blood on a body orifice and her breast examination was also satisfactory.

**Figure 1:**
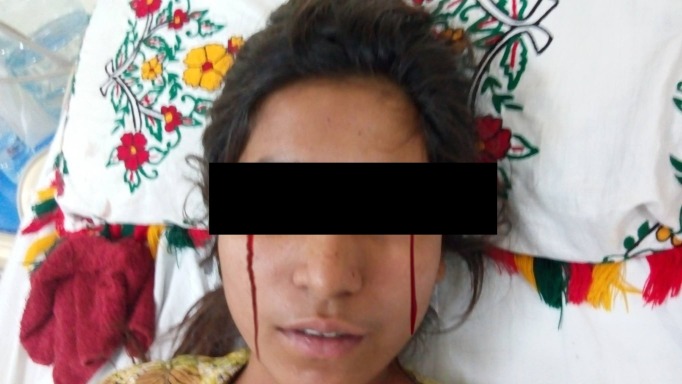
Episode of blood in tears

On her sixth day of admission, the patient complained of continuous chest pain and she said she had it for three to four times per week since last three years. She added that she forgot to address it on the day of admission. Chest pain was sharp in quality and diffuse in nature. ECG had no acute changes. When asked about any previous episodes of chest pain, she told that she had an episode before and that always relieved by getting some fresh air.

On the eighth day of admission, the patient complained about dribbling of urine since eight months. According to her, she used to take bath five times in a day. But on examination, she had not changed her clothes since admission. On observation, she was in acute distress and in severe need of any kind of management. Patient demanded medical or surgical treatment to relieve her different symptoms. She was from the low socioeconomic group and family was concerned about her illness and prompt treatment. They had incurred a huge financial loss for last eight months in seeking medical opinions about her various conditions. She was given different treatments but none of them ever helped.

Such plausible and fabricated medical history raised our concern for a psychiatric consult. After having a long session individually with the patient alone and then with her relatives revealed the fact that she was the only female in her family and therefore; the center of attention and care. Our psychiatrist advised close camera surveillance and vigilant observation of the patient after getting consent from the family. It was found patient used that bottle of blood as a source for her tears. They counseled family members about her symptoms in detail and advised to keep her occupied in different activities with paying less attention to her complaints. A conclusive diagnosis of Munchausen syndrome was made after a detailed assessment. We discharged her after one week and followed-up with serial cognitive behavioral therapy (CBT) sessions.

## Discussion

Munchausen’s Syndrome is a rare psychiatric disorder and a diagnosis of exclusion. In broad term, Munchausen syndrome is a type of factitious disorder with predominantly physical sign and symptoms to seek hospitalization. Such patients are easily consenting to invasive or non-invasive means of treatment. Many patients even demand an invasive intervention. It is always necessary to rule out malingering or any kind of secondary gain other than the hospitalization [3-4].

Another variant described in the literature is Munchausen by proxy in which a caregiver is fabricating symptoms to instigate medical treatment. One should give special attention to this form where falsification and feigning of symptoms might lead to potential harm to the child. This is a form of child abuse and it should be reported to higher officials or child protecting services if mandatory [5-6].

Asher proposed three types of Munchausen presentation; acute abdomen, acute bleeding episode, neurological emergency. However, it may have a variable presentation. Additionally, people employed in the healthcare industry such as nurses, lab technicians, paramedics and their relatives might have an easy access to medications or other materials which may help them to produce symptoms [7-8]. On the other side, genuine illness may coexist in the same patient or new illness may occur in a patient previously diagnosed with Munchausen. Hence, detailed history and physical examination including prompt laboratory evaluation may be mandatory each time because the management would be different [9-10]. Patients undergo various medical and surgical treatments and incur financial loss especially in places where health care is not covered for individuals.

## Conclusions

Patients who might belong to solitary lifestyle or who face sudden cessation of attention from people might feign their symptoms and assume a sick role to get the medical attention. The primary and only intention in such cases is to get the desired level of attention as before. Sometimes patients might go to great lengths to produce symptoms requiring at least an admission in the emergency, if not in medical wards. A diagnosis of Munchausen does not exclude coexistence of a genuine illness and hence detailed physical and prompt evaluation is cardinal before definite diagnosis. Overall, Munchausen and other conditions in the spectrum of factitious disorders remain heavily under-diagnosed by psychiatrists as well as general physicians.
